# Kinetic Modeling of the Assembly, Dynamic Steady State, and Contraction of the FtsZ Ring in Prokaryotic Cytokinesis

**DOI:** 10.1371/journal.pcbi.1000102

**Published:** 2008-07-04

**Authors:** Ivan V. Surovtsev, Jeffrey J. Morgan, Paul A. Lindahl

**Affiliations:** 1 Departments of Chemistry and Biochemistry and Biophysics, Texas A&M University, College Station, Texas, United States of America; 2 Department of Mathematics, University of Houston, Houston, Texas, United States of America; Harvard University, United States of America

## Abstract

Cytokinesis in prokaryotes involves the assembly of a polymeric ring composed of FtsZ protein monomeric units. The Z ring forms at the division plane and is attached to the membrane. After assembly, it maintains a stable yet dynamic steady state. Once induced, the ring contracts and the membrane constricts. In this work, we present a computational deterministic biochemical model exhibiting this behavior. The model is based on biochemical features of FtsZ known from *in vitro* studies, and it quantitatively reproduces relevant *in vitro* data. An essential part of the model is a consideration of interfacial reactions involving the cytosol volume, where monomeric FtsZ is dispersed, and the membrane surface in the cell's mid-zone where the ring is assembled. This approach allows the same chemical model to simulate either *in vitro* or *in vivo* conditions by adjusting only two geometrical parameters. The model includes minimal reactions, components, and assumptions, yet is able to reproduce sought-after *in vivo* behavior, including the rapid assembly of the ring via FtsZ-polymerization, the formation of a dynamic steady state in which GTP hydrolysis leads to the exchange of monomeric subunits between cytoplasm and the ring, and finally the induced contraction of the ring. The model gives a quantitative estimate for coupling between the rate of GTP hydrolysis and of FtsZ subunit turnover between the assembled ring and the cytoplasmic pool as observed. Membrane constriction is chemically driven by the strong tendency of GTP-bound FtsZ to self-assembly. The model suggests a possible mechanism of membrane contraction without a motor protein. The portion of the free energy of GTP hydrolysis released in cyclization is indirectly used in this energetically unfavorable process. The model provides a limit to the mechanistic complexity required to mimic ring behavior, and it highlights the importance of parallel *in vitro* and *in vivo* modeling.

## Author Summary

The division of a cell into two daughter cells is an essential characteristic of living systems. This process requires the assembly of a contractile ring attached to the membrane at the site of division. At the appropriate point of the cell cycle, the ring constricts and pulls the membrane with it, pinching the cell at the division plane and forming daughter cells. In bacteria, the ring is a polymer of the protein FtsZ. The biochemical features of the isolated FtsZ protein have been studied intensively, but how these features are related to the function of the ring in living cells is not clear. In this work, we mathematically modeled the dynamics of FtsZ ring assembly, steady-state maintenance, and contraction both inside and outside of a cellular environment. The model is extremely simple and includes only experimentally known features of the FtsZ protein. The model exhibited behavior associated with the FtsZ ring in living cells, indicating that no additional mechanistic features are needed to explain this behavior. Our systems biology approach has been successful in relating molecular-level chemical processes with cellular-level behavior. A similar approach might be used to develop an analogous model for the more complex contractile rings found in eukaryotic (e.g., human) cells.

## Introduction

One of the fundamental elements of both prokaryotic and eukaryotic cytokinesis is the assembly of a cytoskeletal ring at the future cell division plane. Such rings are attached to the cell membrane and, at the appropriate point in the cell division cycle, contract with the membrane to cause cell division. In prokaryotes, FtsZ is the key protein of this contractile ring. This homologue of the eukaryotic cytoskeletal protein tubulin is among the first proteins to localize at the division plane where it forms the polymeric core of the so-called *Z ring*. At subsequent steps during cytokinesis, at least ten others proteins are recruited to the ring and are involved in contraction in *E. coli*. Homologs of FtsZ appear to be found universally in almost all bacteria and archaea, but this is not the case for other *E. coli* proteins utilized in ring formation. Thus, it appears that the Z ring is a universal component of prokaryotic cytokinesis but that there are species-to-species differences regarding other aspects of the process. Given that an FtsZ homolog is found in wall-less prokaryotes such as *Mycoplasma*, the presence of a cell wall does not appear to be essential for this process. Current knowledge of FtsZ features has been reviewed [1]–[8].

Most biochemical features of FtsZ have been established through *in vitro* experiments. Like tubulin, FtsZ is a GTPase [9], hydrolyzing GTP *in vitro* at a rate of 1–8 GTP molecules per minute per FtsZ monomer [10]–[13]. GTP to GDP hydrolysis occurs at an active site formed by two adjacent FtsZ monomers within a polymer; hence the GTPase activity requires FtsZ polymerization [14],[15]. Polymerization of FtsZ *in vitro* has been studied extensively using sedimentation, light-scattering, fluorescence and EM [3],[6],[9],[16]. When *in vitro* and in the presence of excess GTP, FtsZ self-assembles spontaneously into long single-stranded head-to-tail polymers [12]–[14], [17]–[19]. FtsZ self-assembly appears to be cooperative, as characterized by a time-lag and a critical concentration [16], [19]–[23]. Given that filaments are single-stranded, the mechanism of this apparent cooperativity remains elusive. Although FtsZ predominantly polymerizes into single-stranded filaments, formation of multi-stranded bundles and sheets have been reported [21],[24],[25].

Under some *in vitro* conditions, GDP-bound FtsZ monomers also polymerize, but the equilibrium constant for this process is significantly lower than for GTP-bound polymerization, and observed polymers are relatively short and curved [18],[19]. Also, if assembled FtsZ polymers in a solution containing GTP are exposed to an excess of GDP, they disassemble quickly [26]. Under *in vitro* conditions, greater than 80% of FtsZ subunits within a filaments are GTP-bound [11],[13], in contrast to tubulin filaments. The nearly universal presence of GTP in FtsZ polymer active-sites implies either that GTP can rapidly replace GDP moieties that form within the polymer or that GDP-bound monomers are expelled rapidly from the polymer [13],[14]. Overall, these results cast doubt as to whether GDP-associated polymerization is physiologically relevant [3],[6].

Much less is understood regarding Z ring structure and dynamics *in vivo*. In *E. coli*, the concentration of FtsZ remains nearly constant throughout the cell cycle [27],[28]. Initially FtsZ is dispersed, perhaps as monomers, in the cytoplasm [6]. Near the end of DNA replication, FtsZ localizes to the membrane at midcell in a ring-like structure [29]. Whether the Z ring is a single closed ring, an assembly of closed rings, a helical structure, or a bundle of overlapping open filaments remains unknown [7]. FtsZ is recruited to the membrane via interaction with FtsA and/or ZipA. The MinCDE system and nucleoid occlusion are responsible for the correct localization of the Z ring to the midcell [6],[30]. These factors are crucial for the spatial regulation of FtsZ.

During the cell-cycle, FtsZ rings exhibit three distinctive phases *in vivo*, including ring assembly, dynamic maintenance of the ring, and ring contraction. In *E. coli* cells growing with a 2 hour generation time, the Z ring assembles, once initiated, within 1 minute [31]. The assembled ring is maintained for an extended period (up to 50 minutes [29]). Although the size of the ring appears to be constant, the ring itself is highly dynamic, undergoing fast exchange of subunits with FtsZ in the cytosol [32]–[34]. This exchange occurs with a half-time of 10–30 s as measured by FRAP [32],[33]. The exchange reaction appears to be coupled to GTP hydrolysis, in that the rates of subunit exchange and GTP hydrolysis are both reduced ∼10 fold in a mutant relative to WT levels [32]. Once cell division is initiated, the Z ring constricts over the course of ∼20 minutes [29],[31]. These time intervals place qualitative constraints for kinetic models of *E. coli* cytokinesis (i.e. assembly should be far faster than contraction). The factors that control the timing of ring assembly and the commitment from one phase to another remain unknown, as do the factors that define the kinetics of assembly and contraction.

There are uncertainties as to whether the FtsZ features which are revealed through *in vitro* studies can be applied directly to understanding *in vivo* Z ring behavior [4],[7],[8]. This uncertainty arises, to some extent, by the lack of a kinetic Z ring model capable of assuming both *in vitro* and *in vivo* conditions. So far, only *in vitro* FtsZ polymerization kinetics have been analyzed [12]–[13] and *in vitro* models have been proposed. The kinetic model suggested by Chen and coworkers [20] includes an activation step, a nucleation/dimerization step (with equilibrium constant *K_nuc_*) and a series of favorable elongation steps (up to polymers of length 7, with *K_el_*∼10^2^–10^4^ *K_nuc_*). In the steady state model considered by Gonzalez [35], polymerization was supposed to be isodesmic (i.e. *K_nuc_* = *K_el_*) and, in contrast to the model of Chen *et al.* [20], cyclization of FtsZ polymers was included. The equilibrium constant of cyclization *K_cyc_* was greater than that associated with dimerization and elongation (i.e. *K_cyc_*>*K_el_* [*Fts*Z]). Central to the model of Gonzales *et al.* is the assumption that cyclization will be most probable for polymers of a certain length while less probable for shorter or longer polymers. This is due to the intrinsic curvature of FtsZ polymers which leads to the probability that the ends of polymers of a particular length will be near to each other. The observation of FtsZ rings *in vitro* by EM and AFM [35],[36] demonstrates that cyclization occurs with preferred ring sizes. Proposed models were supported by stopped-flow [20] and sedimentation [35] data and were invoked to interpret the apparent cooperativity of FtsZ assembly. But these models focused only on *in vitro* polymerization. Currently, there is no chemical kinetic Z ring model which would be useful in understanding mechanistically how the FtsZ ring functions *in vivo*. A study describing possible forces involved in contraction has recently appeared [37] but no chemical/molecular aspects of FtsZ contraction were included.

In this study, we have developed a mechanistic model of the **Z ring that uses reactions established by *in vitro* studies and exhibits *in vivo* desired behavior. Such behavior includes assembly, dynamic ring maintenance, and constriction. We have kept the model as simple as possible (i.e. a minimal number of reactions, components and assumptions) and have adhered closely to relevant experimental results. This minimal model semi-quantitatively reproduces the behavior observed for assembly, dynamic stability, and contraction of the Z ring in real cells. Interestingly, this behavior could be reproduced only when interfacial reactions involving the cytosol volume and the membrane surface were assumed. This model limits the mechanistic complexity required to mimic Z ring behavior and it highlights the utility of parallel *in vitro* and *in vivo* modeling.

## Model

In this section we develop a biochemical kinetic model of the FtsZ ring with the behavior described in the *Introduction*. One of the novel aspects of our modeling approach is an ability to consider both *in vivo* and *in vitro* conditions. First, we will consider particular geometrical aspects of the *in vivo* model, followed by a discussion of the known biochemical reactions associated with FtsZ and how these were incorporated into the model. Next, we describe how the assumed geometry and interfacial reactions can influence the kinetics of the system. In the final part of this section, we describe how to transform *in vivo* conditions to *in vitro* conditions, and highlight the advantages of using the same chemical model and parameters to simulate dynamics under both settings.

### Geometrical Considerations

**Figure 1:**
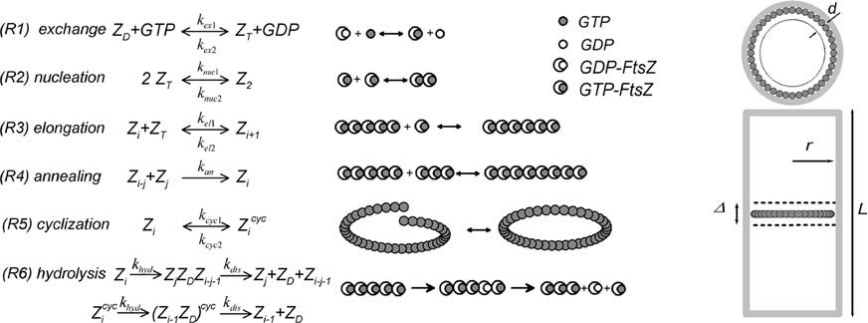
Reactions and Geometry of Model. Z_D_ and Z_T_ indicate GDP- and GTP-bound FtsZ, respectively. Z_i_ and Z_i_^cyc^ indicate polymers of length *i* in open and cyclized forms, respectively. Z_D_, Z_T_, and Z_2_ are assumed to be distributed uniformly in the cytosol of the cell. All FtsZ-polymers starting from trimers and higher are attached to the membrane within the narrow zone Δ. (*R1*) GTP/GTP exchange; (*R2*) nucleation (dimerization) of Z_T_; (*R3*) elongation of FtsZ polymers; (*R4*) annealing of FtsZ polymers; (*R5*) cyclization of FtsZ polymers with open ends; (*R6*) GTP hydrolysis within FtsZ-polymers (both for open and cyclized forms), followed by GDP-FtsZ expulsion and fragmentation of the polymer. Hydrolysis is the rate limiting step, such that intermediates of Reaction *R6* are short-lived and at quasi steady state.

GTP, GDP, GDP-bound FtsZ monomers (abbreviated Z_D_), GTP-bound FtsZ monomers (Z_T_), and FtsZ dimers (Z_2_) are assumed to be distributed uniformly in a well-mixed cytosol. Trimers and all other higher-level open polymers of FtsZ (Z_i_, *i* = 3…*i_max_*) as well as cyclized FtsZ rings are assumed to be attached (by an unspecified mechanism not considered in this model) to the inner membrane surface, specifically within a narrow strip centered at the mid-cell. The mechanism which locates this region is not considered in the model. Membrane-bound FtsZ oligomers are assumed to extend some distance from this surface into the cytosol to give a 3D “reacting zone” (Fig. 1). All surface-bound species are assumed to be well-mixed within the reacting zone. For simplicity, changes in the ratio of the midzone surface area to cytosol volume are not considered.

We describe all cytosolic reagents (GTP, GDP, Z_D_, Z_T_, and Z_2_) by volume concentrations, defined as *n_i_*/*V_cyt_*, where *n_i_* is number of molecules of the reagent and *V_cyt_* is the cytosol volume. Membrane-bound components are described by reduced surface concentrations *n_i_*/(*d S)*, where *S* is the surface area of the reacting zone and *d* is the thickness of that zone. This definition allows the dimensions of rate constants and concentrations for surface and volume reagents to be the same. Thickness *d* is assumed to equal twice the size of an FtsZ protein, i.e. *d* = 8 nm. The width of the reacting zone is assumed to be 2.5% of the length *l* of a cell of radius *r*, such that *S* = 2π *r* 0.025 *L* (we assumed *r* = 0.4 µm and *L* = 4 µm) resulting in surface-to-volume ratio χ = *d S*/*V* = 0.001. The width of the reacting zone is a measure of the precision associated with the localizing mechanism.

### Biochemical Mechanism

The biochemical mechanism assumed by the model includes the following steps, as summarized in Fig. 1.

#### Nucleotide exchange reaction

FtsZ monomers are assumed to exist in two states. Z_T_ (with concentration Z*_T_*) is active towards polymerization, while Z_D_ (with concentration Z*_D_*) is inactive towards polymerization. Z_T_ and Z_D_ interconvert reversibly by exchanging bound nucleotide (Fig. 1, *R1*), in accordance with reaction rates
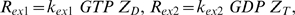
(1)where *k_ex_*_1_ and *k_ex_*_2_ are forward and reverse rate constants, respectively. Considered in the forward direction, this exchange reaction can be viewed as the activation of the monomer toward polymerization. The nucleotide-free state of FtsZ monomers is assumed to be present in insignificant amounts and is not included in the model.

#### Nucleation reaction

According to the model, two Z_T_ monomers can dimerize (Fig. 1, *R2*) with rates
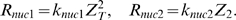
(2)Rate constants for nucleation/dimerization are defined independently of subsequent elongation steps (see *k_el1_* and *k_el2_* below). This allows the model to exhibit either isodesmic or cooperative polymerization depending on whether the specified values for *k_nuc1_* and *k_nuc2_* are the same as or different from *k_el1_* and *k_el2_*, respectively.

#### Elongation

The dimer (Z_2_) and all other *i*-mers (Z_i_) are assumed to elongate incrementally by adding additional Z_T_ monomers (Fig. 1, *R3*), with rates
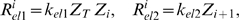
(3)where *R^i^_el_*_1_ and *R^i^_el_*_2_ denote the forward and reverse rates, respectively, for elongation of a polymer of length *i*. To minimize the number of parameters of the model, the rate coefficients for the elongation reactions are assumed not to depend on the length of Z_i_.

#### Annealing of FtsZ polymers

The model includes an annealing reaction between FtsZ polymers (Fig. 1, *R4*) which we consider to be irreversible for simplicity. Similar to elongation reactions, rates of annealing are assumed to be independent of polymer length *i*. Polymers of length *i* result when a polymer of length *j* anneals with a polymer of length *i*–*j*. The resulting positive flux of Z_i_ is the sum of contributions from all possible annealing events:
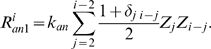
(4a)Here the Kronecker delta *δ_i i_*_–*j*_ renders the coefficient in the summation equal to 1 for the annealing of polymers of the same length (*j* = *i*/2) and equal to 1/2 for the annealing of polymers with different length (*j*≠*i*/2). Doing this avoids counting the same event twice. Simultaneously, polymers of length *i* disappear due to their annealing with another polymer. The resulting negative flux of Z_i_ is
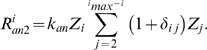
(4b)Here *i_max_* is the maximum length of polymer considered in the model.

#### Cyclization of open polymers

Reactions 1–4 provide a mechanism for FtsZ polymers to assemble. Following Gonzales *et al.* [35], Z_i_ polymers are assumed to have an intrinsic curvature, which leads to the curved shape of FtsZ-polymers. This intrinsic curvature leads to a non-zero probability for polymer cyclization (Fig. 1, *R5*), which is considered as a reversible process with rate constants *k_cyc_*_1_ and *k_cyc_*_2_, and reaction rates
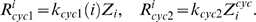
(5a)The dependence of rate constant *k_cyc1_* on polymer length *i* is an important feature of the model. Cyclization involves two open ends reacting, suggesting that the probability of this reaction should depend on the distance between the ends of the polymer, and thus on length *i*. We assume that the reverse reaction (de-cyclization) does not depend on polymer length for simplicity. As such, we consider that *k_cyc1_* depends on the length of Z_i_, with the optimal rate occurring at *i*_0_, given by
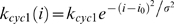
(5b)where σ represents the width of the distribution of rates. This equation implies that the chemical potential is symmetric about a minimum at *i*_0_ (resulting in a maximum for Eq. 5b). For free polymers unattached to the membrane, *i*_0_ is determined by the intrinsic curvature of the polymers. This intrinsic curvature is assumed to be sufficiently flexible such that curvature of different radii can be realized. Indeed, rings of different lengths are observed *in vitro* by electron and atomic force microscopy [35],[36]. For open polymers attached to the membrane, membrane circumference dictates the curvature of these polymers and hence the optimal length *i*_0_ for an open polymer to cyclize.

#### Cyclization after ring assembly

According to our model, once one or more membrane-anchored Z rings have assembled, membrane circumference at midcell is no longer free to change. This situation causes the “tables to turn”, so to speak, in that the average ring length now dictates membrane circumference, which in turn defines *i*_0_, the optimal length of an open polymer to cyclization. We model this situation using the same Eq. 5b to describe *k_cyc1_*(*i*), but with *i*_0_ set equal to the current average ring size, given by
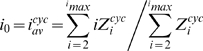
(5c)The assumption that 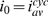 makes the cyclization of polymers self-regulating, and it provides a mechanism by which the size of Z rings can vary.

#### GTP hydrolysis in polymers

According to the model, the hydrolysis of GTP within an open polymer leads to the formation of an intermediate designated {Z_j_Z_D_Z_i−j−1_} (Fig. 1, *R6*). This intermediate is assumed to be unstable, congruent with our assumption that Z_D_ units are incapable of polymerizing. Thus, the Z_D_ unit in such intermediates is assumed to be expelled, fragmenting the polymer in the process. This fragmentation leads to two shorter open polymers. For an initially cyclized polymer, GTP hydrolysis/fragmentation would result in a single open polymer. The first step of the process, namely GTP hydrolysis, is assumed to be rate-limiting, while the second step (Z_D_ expulsion and fragmentation) is assumed to be fast. Hence, we assume a quasi-steady state condition for the Z_j_Z_D_Z_i−j−1_ intermediate, namely d{Z*_j_*Z*_D_*Z*_i_*_−*j*−*1*_}/d*t*≈0. This allows us to substitute Z_j_Z_D_Z_i__−j−1_ concentrations by constant algebraic expressions in the ODEs associated with the model. The result is an effective reaction where hydrolysis, polymer fragmentation, and Z_D_ expulsion occur simultaneously in a single step with the following rates for open and cyclized polymers, respectively:
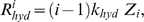
(6a)

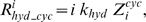
(6b)This reaction occurs for both open and cyclized polymers, as there is no obvious means by which an active-site for GTP hydrolysis could “know” whether it was part of an open or cyclized polymer. So, we assume that the process occurs at the same rate on a per monomer basis. Eq. 6a and 6b specify that the rate of hydrolysis is proportional to the number of active sites in the polymer, namely *i*−1 and *i* for open and cyclized polymers, respectively.

For an open polymer of length *i*, hydrolysis results in two shorter polymers Z*_j_* and Z*_i_*_−*j*−1_, with the values of *j* and *i*−*j*−1 dictated by the position of the hydrolysis event. Hydrolysis and subsequent breakage will occur with the same probability at any of the (*i*−1) active sites. The probability that a polymer of length *j* exists among the products will be 1/(*i*−1) for the *j* = *i*−1 event (this event leads to the formation of Z_i__−1_ and Z_D_) and 2/(*i*−1) for any *j*<*i*−1. Appropriate terms of the kinetic equations were multiplied by these coefficients to take these probabilities into account in determining the different possible products of the reaction. For a cyclized polymer, the product of the hydrolysis and the following expulsion are always the same – namely Z_D_ and an open polymer of length *i*−1.

### Interfacial Reactions

An inevitable issue of modeling Z ring dynamics *in vivo* is whether to consider interfacial reactions between a 3D volume such as the cytosol of the cell, and the 2D membrane at which the ring assembles. In our model, dimerization and elongation of the dimer to form a trimer are assumed to occur in the cytosol. Elongation of trimers and higher polymers are assumed to occur on the interface between the membrane where the polymers are localized and the cytosol where monomers are localized. Hence, these processes are interfacial. Conservation of matter requires that fluxes of reactants of such interfacial reactions be balanced by including the surface-to-volume ratio (χ) of the reacting system in the kinetic equations [38]. This requirement was satisfied by multiplying or dividing particular terms within the system of differential equations with the dimensionless surface-to-volume ratio defined as χ = *d S*/*V_cyt_*. For example, the reaction Z_i−1_+Z_T_→Z_i_ has fluxes balanced as 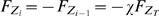.

Note that GTP hydrolysis is also an interfacial reaction (except for the hydrolysis occurring in the dimer) since polymers locate on the membrane surface while Z_D_ is in cytoplasm. Similarly, annealing of a dimer to a polymer is an interfacial reaction, since the reacting dimer is in the cytosol while the polymer is on the membrane. Annealing and cyclization of higher polymers are entirely surface processes and thus do not require any modification of kinetic terms. For simplicity we assume that values of the rate constant for annealing and elongation are the same in the cytosol and on the surface.

### *In Vivo* versus *In Vitro* Modeling

Interfacial reactions would take place only under *in vivo* conditions, when the membrane and membrane-bound species are present. Within our framework, an *in vivo* model can be transformed into an *in vitro* model simply by assuming χ = 1, as this returns the ODEs into those generated using the law of mass action. A more subtle difference between *in vitro* and *in vivo* modeling has to do with the assumed distribution for the cyclization rate constant *k_cyc1_*(*i*). Under *in vivo* conditions, this distribution should be narrow because the curvature of the polymer will follow the membrane circumference closely. Under *in vitro* conditions, this distribution should be broad as there is no membrane template and FtsZ polymers are assumed to be flexible.

### Numerical Simulations

The model reactions of Fig. 1 were used to generate the set of ODEs, with reaction rates indicated in Eq. 1–6.
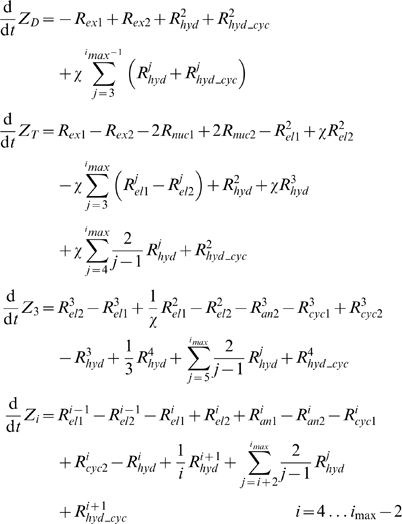
(7)

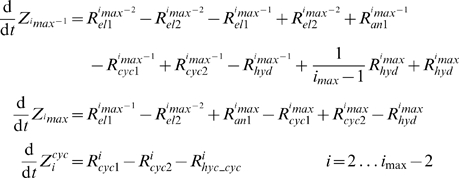
Appropriate terms include χ in Eqs. 7 as discussed above. For simplicity, *GTP* and *GDP* were assumed to remain constant, emulating an *in vitro* situation where GTP is in excess and an *in vivo* situation where the cell homeostatically maintains a particular *GTP*/*GDP* concentration ratio. The maximum length of polymers considered in our calculations was *i_max_* = 150, resulting in 300 ODE's describing the concentrations of open and cyclized forms of polymers of different lengths. The system of ODEs was numerically solved using XPP-AUTO software [39]. We chose a value of χ = 0.001 based on an estimate of the cell-cycle averaged geometry for the cell in Fig. 1. The average length of open polymers at time *t* was calculated as
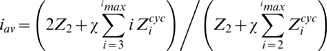
(8)The average ring size was calculated using Eq. 5c. In simulating the assembly kinetics of the FtsZ mutant L68W, the fluorescent signal was calculated as
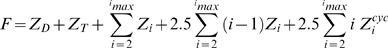
(9)assuming a 2.5-fold enhancement of fluorescence at the interface of adjacent monomers [20].

### Selection of Rate Constants

Rate constants for simulations were selected according to the following considerations. We assumed that *K_ex_* = 2 based on the values *K_D_* = 4 µM and *K_D_* = 8 µM reported by Mukherjee *et al.* for GTP and GDP binding to FtsZ, respectively [40]. Chen *et. al* measured ∼1 s^−1^ for the apparent first-order activation of FtsZ [20]. The rate for this process in our model equals *k_ex1_ GTP* Z*_D_* so for an assumed *GTP*≈100 µM, we obtain *k_ex_*_1_≈0.01 µM^−1^ s^−1^. The rate constant for the reverse exchange reaction was then assumed to be *k_ex_*_2_ = 0.005 µM^−1^ s^−1^.

Rate constants selected for the elongation reaction (*k_el_*_1_ = 4 µM^−1^ s^−1^, *k_el_*_2_ = 0.4 s^−1^) were those reported for a stopped-flow study of FtsZ polymerization in a physiological buffer [20]. In the same work, Chen and coworkers [20] reported a rate constant for the forward nucleation reaction of *k_nuc1_*≈4 µM^−1^ s^−1^. A wide range of rate constants for the reverse nucleation reaction was also reported, depending on the buffer used [20]; we selected an intermediate value of *k_nuc_*_2_ = 40 s^−1^.

Rate constants for the cyclization reaction have not been measured experimentally. For simplicity, the rate constant for decyclization was assumed to be the same as that for depolymerization (i.e. *k_cyc_*_2_ = *k_el_*_2_ = 0.4 s^−1^). Gonzalez *et al* [35] suggested that the observed sedimentation distribution of FtsZ polymers could be explained by assuming that the equilibrium constant *K_cyc_* was higher than *K_el_* ([FtsZ] *K_el_*/*K_cyc_* = 0.01–1). In our simulations we used *k_cyc_*_1_ = 60 s^−1^ (which affords the relationship [FtsZ] *K_el_*/*K_cyc_* = 0.7). Using higher *k_cyc1_* values did not yield a stable steady state of assembled FtsZ rings (see below).

Although annealing was mentioned several times as a potential process occurring during Z ring assembly [8] it has not been included in any model of FtsZ polymerization, and no experimental values have been reported. For simplicity we assumed that annealing was irreversible with *k_an_* = *k_el_*_1_ = 4 µM^−1^ s^−1^. Finally, we assumed *k_hyd_* = 0.15 s^−1^ based on the value reported by Romberg and Mitchison [13].

## Results

Numerical integration of the model developed above can semi-quantitatively reproduce the *in vivo* behavior sought in this study. This behavior includes the assembly of the Z ring via polymerization followed by cyclization, a “dynamic steady state” in which rings appear stable (in terms of concentrations and sizes) but hydrolysis continuously leads to the exchange of monomeric subunits between cytosol and the ring, and finally the contraction of the ring. Transitions between these phases are not included in the model as they undoubtedly involve cellular regulatory aspects that are not currently understood. By adjusting just two parameters (χ and σ), the same chemical model can simulate *in vitro* conditions.

We begin with the *in vitro* self-assembly of the FtsZ filaments, as this has been studied most experimentally. Then we switch to *in vivo* conditions, where FtsZ polymerization results in Z ring assembly. This is followed by an exploration of the dynamic steady state in which a self-regulating ring is maintained indefinitely. Finally, we show how a hypothetical triggering stimulus can induce the constriction of the ring, and we characterize the kinetics of contraction.

### *In Vitro* Assembly of the FtsZ Ring

To mimic *in vitro* conditions, we set χ = 1 in the ODEs of Eq. 7, σ = 10 FtsZ units in Eq. 5b, and *i_0_* = 100. With χ set to 1, the ODE's become the standard set which would be generated, using the law of mass action, from the set of chemical reactions associated with the model of Fig. 1. The chosen σ and *i*_0_ values reflect the distribution and mean ring circumferences observed by Gonzalez *et.al* [35] at high surface occupancy, but they also reflect the practical limitations of our simulations which requires *i_max_* = 150. We chose *i_0_* = 100 to avoid simulation artifacts that could arise with *i_0_* values chosen closer that limiting value.

**Figure 2:**
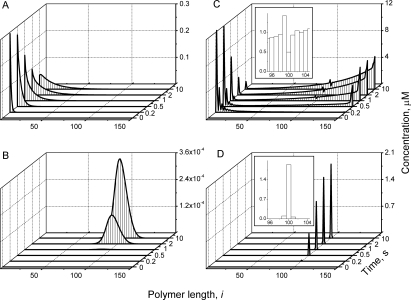
Dynamics of FtsZ Polymers Distribution during Assembly. Polymer length distributions are plotted as the concentration of polymers of particular length *Z_i_ vs.* length *i* at different time points. (*A*) Open polymers, and (*B*) cyclized polymers under *in vitro* conditions with χ = 1 and σ = 10. (*C*) Open polymers, and (*D*) cyclized polymers under *in vivo* conditions with χ = 0.001 and σ = 0.5. Insets show the polymer length distribution at the final time (30 s) around *i*_0_ = 100. Other parameters: *k_ex_*_1_ = 0.01 µM^−1^ s^−1^, *k_ex_*_2_ = 0.005 µM^−1^ s^−1^, *k_nuc_*_1_ = 4 µM^−1^ s^−1^, *k_nuc_*_2_ = 40 s^−1^, *k_el_*_1_ = 4 µM^−1^ s^−1^, *k_el_*_2_ = 0.4 s^−1^, *k_an_* = 4 µM^−1^ s^−1^, *k_cyc_*_1_ = 60 s^−1^, *k_cyc_*_2_ = 0.4 s^−1^, *k_hyd_* = 0.15 s^−1^, *i*_0_ = 100, *Z_tot_* = 20 µM, *GTP* = 90 µM, *GDP* = 10 µM.

Our initial simulation assumed that an excess of GTP is reacted with a solution of unactivated FtsZ monomers, affording total *Fts*Z, *GTP* and *GDP* of 20, 90, and 10 µM, respectively. Polymerization yielded the distributions of open and cyclized polymers shown in Fig. 2 *A*, and *B*, respectively, plotted as the concentrations of polymers Z*_i_* versus the length *i* of those polymers. Although very long open polymers formed (up to *i_max_*), the distribution at *t* = 10 s includes a majority of relatively short open polymers (*i*<30) with *i_av_*≈13. Cyclized polymers also formed, but with negligible concentrations (Fig. 2 *B*). The extremely low concentration of rings is due mainly to the absence of long open polymers, the reaction-precursors of these rings. The resulting distribution of cyclized polymers is centered at *i*≈90, which differs from the assumed optimal cyclization length of *i_0_* = 100. One factor that gives rise to this shift is the decline in the concentration of open polymers as polymers lengthen. Another factor is that the rings undergo GTP hydrolysis followed by ring opening (Fig. 1 *R6*). This depletes bigger rings and repletes shorter ones, biasing the system toward smaller-sized rings.

**Figure 3:**
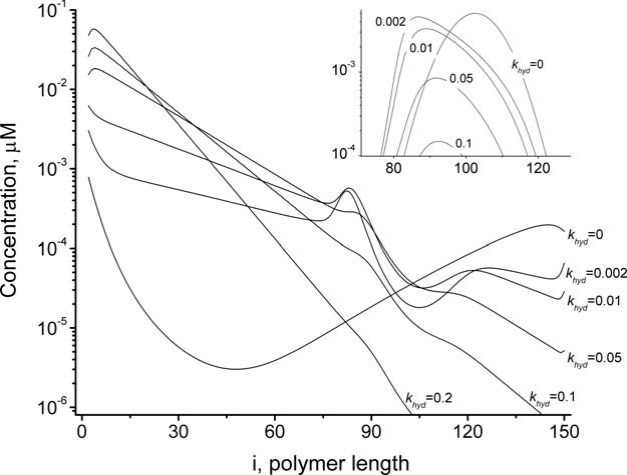
Influence of GTP Hydrolysis Reaction on FtsZ Polymer Distributions *In Vitro.* The concentrations of open polymers of lengths *Z_i_* at the final time (30 s) are plotted as functions of length *i*. *Inset* shows the distribution for cyclized polymers. Parameters are the same as in Fig. 2 *A*–*B* except that *Z_tot_* = 10 µM, *k_hyd_* = 0, 0.002, 0.01, 0.05, 0.1, 0.2 s^−1^ for different simulations, as shown.

In contrast to other *in vitro* models of FtsZ assembly, our model includes hydrolysis and annealing reactions. The effect of hydrolysis is minor for short polymers (because *k_hyd_*<<*k_el_*_1_). The influence of hydrolysis becomes increasingly important as polymers lengthen, because the rate of hydrolysis on a per polymer basis increases with polymer length (Eq. 6). Without hydrolysis (*k_hyd_* = 0), cyclized polymers dominate, with significant fractions of short and long open polymers (Fig. 3 and Fig. S1). In the case of *k_hyd_* = 0.0 the dependence of the polymer distribution on total concentration of FtsZ (Fig. S1 *B*) is similar to that which Gonzalez *et al.* observed by ultracentrifugation [35]. Increasing hydrolysis rates shortened polymers and made the distribution closer to exponential, as evidenced by the decrease in the (log-scale) slope of the distribution (Fig. 3). Hydrolysis also decreased the fraction of cyclized polymers (Fig. 3 *inset*).

**Figure 4:**
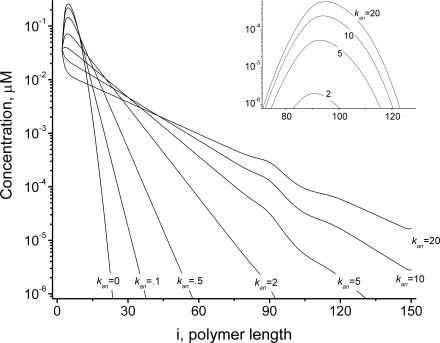
Influence of the Annealing Reaction on the FtsZ Polymer Distributions *In Vitro.* The concentrations of polymers of lengths *Z_i_* at the final time (30 s) are plotted as functions of length *i*. *Inset* shows the distribution for cyclized polymers. Parameters are the same as in Fig. 2 *A*–*B* except that *Z_tot_* = 10 µM, *k_an_* = 0, 0.1, 0.5, 2, 5, 10, and 20 µM^−1^ s^−1^ as shown.

The annealing reaction causes long polymers to accumulate since it decreases the number of nucleated chains and counterbalances the fragmentation associated with hydrolysis. Fig. 4 indicates the dependence of the final polymer distribution (at the end of assembly) on the rate of annealing. In the absence of annealing, short polymers dominate. Increasing the annealing rate spreads this distribution out and shifts it to longer polymers. The Fig. 4 inset shows that faster annealing results in higher concentrations of cyclized polymers. A 2-fold increase in annealing rate (from 5 to 10 µM^−1^ s^−1^) results in a 3-fold increase in the concentration of the most abundant cyclized polymer.

**Figure 5:**
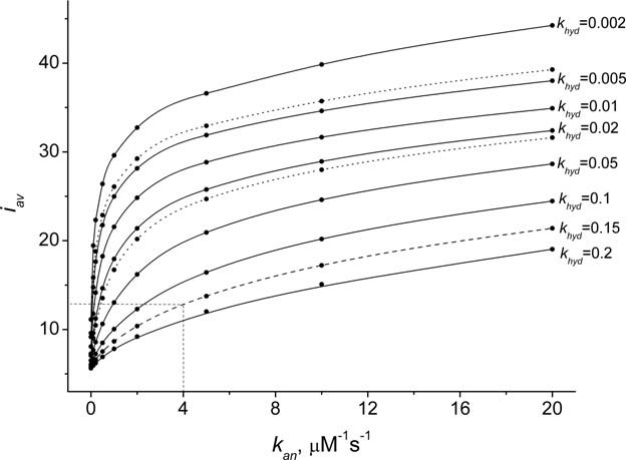
Dependence of FtsZ Polymer Length on Polymers Annealing and GTP-Hydrolysis *In Vitro.* Average length of polymers *i_av_ vs. k_an_* for *k_hyd_* = 0.002, 0.005, 0.01, 0.02, 0.05, 0.1, 0.15, 0.2 s^−1^ and *GTP* = 99 µM, *GDP* = 1 µM. Remaining parameters are the same as in Fig. 4. Dashed lines represent parameters used for *in vivo* simulations (*k_an_* = 4 µM^−1^ s^−1^, *k_hyd_* = 0.15 s^−1^, *i_av_* = 12.8). Dotted lines are for *k_hyd_* = 0.004 s^−1^ (upper curve) and *k_hyd_* = 0.025 s^−1^ (lower curve), reported in [12].

Fig. 5 illustrates how the *average* length of open polymers *i_av_* obtained at the end of the assembly process varies with annealing and hydrolysis rate-constants. The dashed curve and lines represent the hydrolysis and annealing rates (*k_hyd_* = 0.15 s^−1^ and *k_an_* = 4 µM^−1^ s^−1^, resulting in *i_av_* = 12.8) used in most of our simulations. Without annealing (and keeping the remaining rate-constants fixed) *i_av_* declines from about 13 to 6 monomers, revealing the importance of this process. Dotted curves represent the dependence of averaged polymer length for *k_hyd_* = 0.025 and 0.004 s^−1^ (values reported in [12] for GTP hydrolysis and for the hydrolysis of the slow-hydrolyzing GTP analog guanylyl-(α,β)-methylenediphosphonate, respectively). With *k_an_* = 4 µM^−1^ s^−1^, simulations resulted in *i_av_*≈23 and 34, respectively. These predicted average polymer lengths are close to 23 and 38, as determined by electron microscopy using [FtsZ] = 3.5 µM [12]. Although this is lower then the 10 µM concentration used in our calculations, the authors reported no apparent dependence of their results on FtsZ concentration. Using total [FtsZ] = 3.5 µM in our simulations altered *i_av_* to 18 and 26, respectively, which deviates somewhat from experimental results but the ratio of lengths is approximately that observed. Thus, our model semi-quantitatively predicts the effect of hydrolysis rate on polymer length.

**Figure 6:**
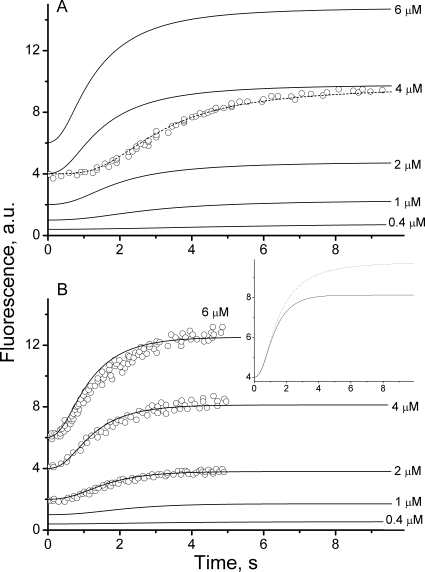
Simulation of Stopped-Flow Fluorescence Experiments by Chen *et al.* Using the FtsZ L68W Mutant [20]. Simulated fluorescence (calculated as in Eq. 9) plotted *vs.* time. (*A*) *k_hyd_* = 0.0 s^−1^; (*B*) *k_hyd_* = 0.15 s^−1^. Total FtsZ concentration is given for each curve. *GTP* = 99 µM, *GDP* = 1 µM. Remaining parameters are as in Fig. 2 *A*–*B*. Inset compares overlapped traces for *FtsZ* = 4 µM and *k_hyd_* = 0.0 s^−1^ (*dashed*) and *k_hyd_* = 0.15 s^−1^ (*solid*). Circles represent experimental data for *FtsZ* = 2, 4, 6 µM (*panel B*) and *FtsZ* = 3.9 µM (*panel A*) taken from [20]. Dashed line in A is a simulation using *k_nuc2_* = 4000 s^−1^, and all other parameters unchanged.

**Figure 7:**
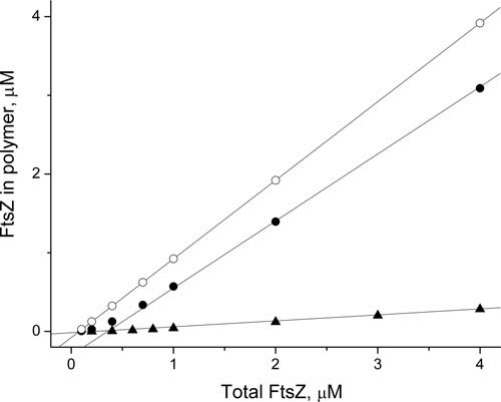
Dependence of FtsZ Assembly on Total FtsZ Concentration. *Open circles*, *k_hyd_* = 0.0 s^−1^ and *in vitro* conditions (χ = 1, σ = 10); *Closed circles*, *k_hyd_* = 0.15 s^−1^ and *in vitro* conditions; *Triangles*, *k_hyd_* = 0.15 s^−1^ and i*n vivo* conditions (χ = 0.0015, σ = 0.5). Remaining parameters are as in Fig. 2. Lines represent best-fit linear approximations which resulted in critical concentrations of 0.08 µM (*in vitro*, *k_hyd_* = 0.0 s^−1^), 0.36 µM (*in vitro*, *k_hyd_* = 0.15 s^−1^), and 0.22 µM (*in vivo*, *k_hyd_* = 0.15 s^−1^).

Our model was used to simulate the stopped-flow kinetics of FtsZ assembly as measured by Chen and coworkers using the FtsZ-L68W mutant [20]. They found that fluorescence was enhanced when monomer-monomer interfaces formed during polymerization. Z_D_ monomers were reacted against GTP in buffer that either contained or did not contain Mg^2+^ ions. The presence of Mg^2+^ ions activated the GTP hydrolysis reaction [3],[20]. Using our model, these experiments could be simulated by assuming different rates of hydrolysis. Time profiles of the simulated fluorescence intensity without (*k_hyd_* = 0.0 s^−1^) and with (*k_hyd_* = 0.15 s^−1^) GTP hydrolysis are shown in Fig. 6 *A* and *B*, respectively. Our model reproduced the observed dependence of both the kinetics of polymerization and steady state levels on FtsZ concentration. Simulations mimicked experimental traces with high fidelity for the Mg-replete buffer (*k_hyd_* = 0.15 s^−1^), including a similar initial lag phase, a similar development of fluorescence, and a similar FtsZ-dependence (Fig. 6 *B*, circles). For Mg-deplete buffer, simulations did not include the elongated lag period reported by Chen *et al* (Fig. 6 *A*, circles), but they did show a slower approach to steady state (Fig. 6 *inset*), in qualitative agreement with their experimental results. The elongated lag phase could be simulated (Fig. 6 *A*, dashed line) with the model by increasing the reverse nucleation rate-constant *k_nuc2_* 100-fold (similar to approach taken in [20]). This suggests that Mg^2+^ ions might somehow be involved in stabilizating the dimer, perhaps by increasing monomer-monomer interaction at the interface within the dimer. A critical concentration of FtsZ is required for polymerization to commence, suggesting cooperativity [3], [6]–[8]. Simulations obtained by our model exhibited similar behavior. In Fig. 7, the concentration of FtsZ found in polymerized forms (i.e. the degree of polymerization) after assembly is plotted as function of total *Fts*Z. Closed circles represent data for *k_hyd_* = 0.15 s^−1^ while open circles represent data for *k_hyd_* = 0.0 s^−1^. Best-fit lines to the *in vitro* data points indicate critical concentrations of 0.36 and 0.08 µM, respectively. A wide range of critical concentrations have been reported, depending on experimental conditions (e,g. pH, Mg and K ion concentrations), including 0.31 µM under GTP-hydrolyzing conditions [22]. Using GTP and a slow-hydrolyzing GTP adduct, critical concentrations of 1 and 0.7 µM, respectively, were obtained [21]. A similar influence of GTP hydrolysis was observed using FtsZ from *Methanococcus jannaschii* [22]. These studies indicate the same effect of GTP hydrolysis as we observe.

### *In Vivo* Assembly of the FtsZ Ring

As discussed in *Model*, we mimicked *in vivo* conditions by setting χ = 0.001 and σ = 0.5. This value of χ restricts surface-bound reactions to the reacting zone centered at the mid-cell, while this value of σ restricts cyclization reactions almost exclusively to those involving polymers of length *i*_0_−1, *i*_0_, and *i*_0_+1. To better compare *in vitro* vs. *in vivo* conditions, we set *i*_0_ = 100 and assumed the same set of reactions and kinetic parameters and concentrations as those used for *in vitro* conditions. Overall *in vivo* FtsZ concentrations ranging from 5 to 20 µM have been reported [7],[8]; we used 20 µM for the majority of our simulations. Similarly, we used *GTP* = 90 and *GDP* = 10 µM in most of our *in vitro* and *in vivo* simulations; these values are close to the experimental *GTP*/*GDP* ratio of 7∶1 reported for *S. typhimurium* [41].

The resulting polymer distributions for open and cyclized forms of FtsZ at different times during *in vivo* assembly are shown in Fig. 2 *C* and *D*. Although only two parameters (χ and σ) differ from the *in vitro* assembly simulation shown in Fig. 2 *A* and *B*, the resulting distributions during *in vivo* assembly differed substantially, in that longer open polymers dominated (Fig. 2 *C*) and a significant fraction of cyclized polymers appeared (Fig. 2 *D*). The total concentration of cyclized polymers (Z*^cyc^* = 2.1 µM) is far greater than under *in vitro* conditions (Z*^cyc^* = 0.006 µM), because the concentrations of long open polymers are much higher. Also, under *in vivo* conditions, cyclized polymers with a very narrow range of circumferences are formed, including only rings with *i* = 99, 100 and 101 (Fig. 2 *D inset*). The most abundant form has *i* = 100, as dictated by the assumed values for *i_0_* and σ (Eq. 5b). Intense cyclization for *i* = 100 depletes Z_100_ polymers (Fig. 2 *C inset*), the substrate for this reaction, and it enriches Z_99_ polymers, the product of the GTP hydrolysis of Z^cyc^_100_ rings. Increased *Z_99_* causes the observed asymmetrical distribution of Z*^cyc^_i_* (Fig. 2 *D inset*).

Higher concentrations of long open polymers (*i_av_*≈63) are also evident for *in vivo* simulations, which leads to higher concentrations of rings. This difference is the result of applying a small χ to the ODEs (Eq. 7), as doing so increases the effective FtsZ concentration at the midzone surface; i.e. small changes of *Fts*Z in the cytosol result in large changes in surface concentrations. The fraction of FtsZ that cyclizes depends on the total concentration of FtsZ present in the system (Fig. 7 *triangles*). Linear approximation results in an apparent critical concentrations of *Fts*Z = 0.22 µM for ring assembly under these conditions.

**Figure 8:**
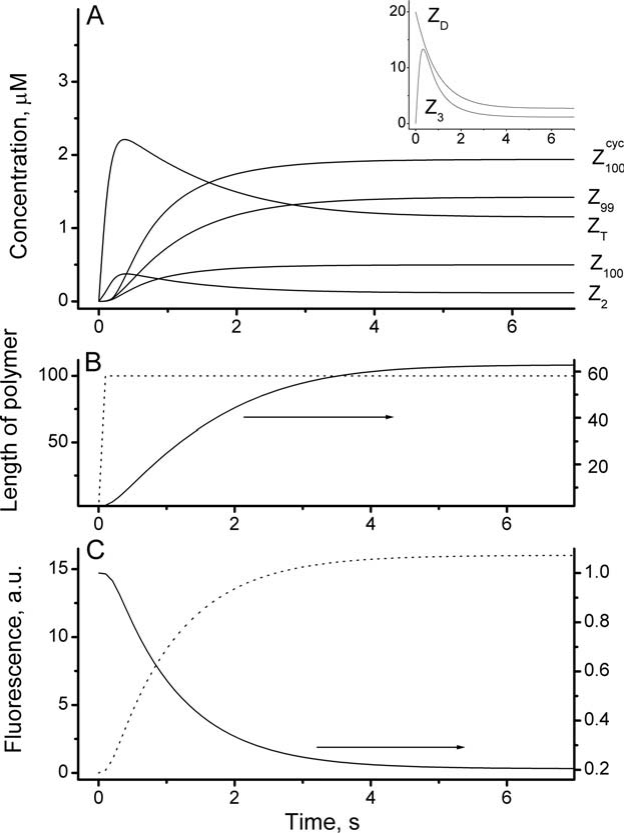
Kinetics of Z Ring Assembly *In Vivo.* (*A*) Concentration of selected polymers *vs.* time. (*B*) Average length of open (*solid*) and cyclized (*dotted*) polymers *vs.* time. (*C*) Fluorescence of the ring-like structure *vs.* time: *dotted* – total concentration of membrane-bound FtsZ polymers (open plus cyclized polymers); *solid* - fraction of FtsZ in cytoplasm. These fluorescence profiles represent a prediction of the model that might be measurable in experiments. Parameters are the same as in Fig. 2 *C* and *D*.

Despite differences in steady state polymer distributions, the time-scale of *in vivo* ring assembly is similar to those obtained under *in vitro* conditions, in that assembly is complete ∼10 sec after the process initiates. Fig. 8 *A* shows time profiles for the formation of various polymer species during *in vivo* assembly. Z_D_ rapidly declines from its initial concentration (20 µM) as various intermediates (e.g. Z_T_, Z_2_, Z_3_) develop and then decline with time. Starting from dimer Z_2_, there is a noticeable time-lag in the kinetics, which depends on the size of polymer. Longer polymers (Z_99_, Z_100_) exhibit longer lags, but remain at significant concentrations as steady state approaches. Cyclized forms (e.g. Z^cyc^_100_ in Fig. 8 *A*) have kinetics similar to open forms. The average length of open polymers continuously grows to a maximum of ∼60 subunits within ∼5 sec (Fig. 8 *B*, *solid line*) while the average circumference for cyclized polymers (Fig. 8 *B*, *dotted line*) develops almost instantaneously to ∼100 and remains at this circumference throughout the assembly process. In contrast, the *concentrations* of individual cyclized polymers (Fig. 2 *D*) develop gradually over the first ∼5 sec of the assembly period, similar to the situation with open polymers.

By comparing the FtsZ-GFP fluorescence due to ring-like structures and that due to the background fluorescence in the cytosol, Sticker *et al* [32] estimated that ∼60% of FtsZ molecules in most (and 30% of FtsZ molecules in some) cells are located in the cytosol at the end of ring assembly. According to our model, all trimers and longer polymers (no matter whether they are in an open or cyclized conformation) localized to the mid-zone surface, and (if properly labeled) would contribute to ring fluorescence at microscopic resolution. Assuming this, our model estimates that cells would contain 20% of FtsZ molecules in the cytosol for the conditions of Fig. 2 *C* and *D*, in reasonable qualitative agreement with the experimentally determined values. The discrepancy might be due to the simplicity of our model or to use of GFP-tagged FtsZ as expressed by the plasmid employed (Wu and Pollard observed a difference in YFP-actin localization compared to that of native actin [42]).

Sun and Margolin have determined that in *E coli* cells growing with a generation time of 2 hrs, the Z ring assembles within 1 min; however the time-resolution of their study was insufficient to reveal the detailed kinetics of this process [31]. Our model can predict the detailed kinetics of the *in vivo* development of ring fluorescence, including the absolute fluorescence signal in the mid-zone (Fig. 8 *C dotted line*) and the time-dependent FtsZ distribution between cytosol and membrane during ring assembly (Fig. 8 *C*, *solid line*).

### Dynamic Steady State of the Ring

As long as the optimal ring size is dictated by membrane circumference (i.e. fixed *i*_0_ = 100), the system of ODEs (Eq. 7) is attracted to a stable steady state in which Z rings with *i*∼100 are assembled. Once the ring(s) is(are) assembled, the membrane circumference should always match the Z ring size; i.e. the membrane circumference and ring size become interconnected. We represent this by applying Eq. 5c, which assumes that membrane circumference (and thus, optimal ring size *i*_0_) equals the average size of the rings *i^cyc^_av_*. This makes the ring size self-regulated, since changes in this parameter will cause changes in *i*_0_ and, simultaneously, *i*_0_ determines the most abundant cyclized form.

**Figure 9:**
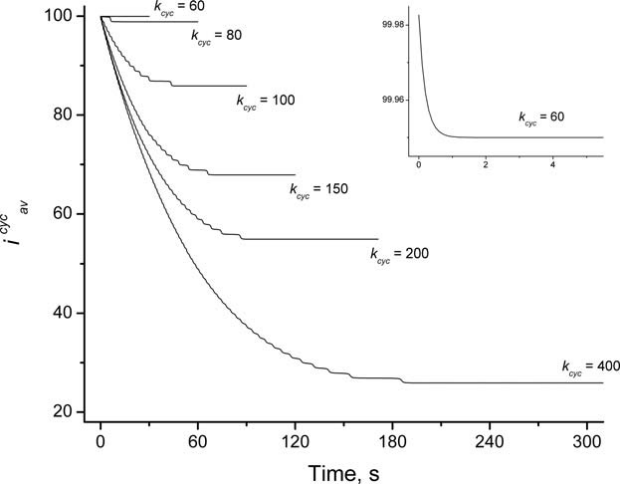
Dynamics of Z Ring after Assembly. The average circumference of Z rings (*i^cyc^_av_*) is plotted as a function of time. Stability of the ring depends on the rate of cyclization, as indicate in units of s^−1^. Remaining parameters are as in Fig. 2 *C*–*D*. Values at final point of assembly (*t* = 30 s, calculated as in Fig. 2 with *i*_0_ = 100) were used as initial values. Starting at time *t* = 0, *i*_0_ was set equal to *i^cyc^_av_* (Eq. 5c).

Fig. 9 shows the dynamics of the assembled ring after Eq. 5c has been applied. With ring-size now free to vary, the system readjusts to a new steady state that differs from the original steady state to a degree that depends non-intuitively on the rate of cyclization. With *k_cyc_* = 60 s^−1^, virtually no spontaneous contraction occurred (the inset of Fig. 9 reveals a very slight contraction) such that there was little difference between new and original steady states in terms of ring size. At faster rates of cyclization, the initial spontaneous contraction was more severe. In all cases, concentrations of almost all open polymers did not change significantly, even when the average ring size did change (Fig. S2).

*In vivo* experiments show that the assembled Z ring can be stable for a long period [29],[31]. Given the results of Fig. 9, we selected a rate constant of cyclization ≤*k_cyc1_*∼60 s^−1^. Stability also depended on hydrolysis and polymerization rates (data not shown). As expected, fast polymerization rates maintained stable average ring size while slow polymerization rates did not. The influence of hydrolysis rates was less intuitive. Increasing this rate helped maintain a stable average ring size, while lowering the hydrolysis rate caused instability. Other factors that influenced ring size stability included the width of the distribution for cyclization rates (narrower improved stability but hindered contraction) and the total FtsZ concentration (within some range, higher concentrations improved stability).

FRAP (Fluorescence Recovery After Photobleaching) experiments by Erickson and co-workers have established that the Z ring is dynamic, in that cytoplasmic FtsZ rapidly replaces (photobleached) subunits of the Z ring [32],[33]. For WT cells, a half-time of ∼30 seconds for fluorescence recovery was reported [32]. Importantly, this rate was coupled with the rate of GTP hydrolysis. Sticker *et al.* observed that a 10-fold decline in GTP hydrolysis by the FtsZ84 mutant was associated with a 9-fold decline in the rate of FtsZ monomer turnover [32]. At dynamic steady state, the rate by which FtsZ monomers are incorporated into the ring equals the rate at which FtsZ monomers dissociate from the ring, since incoming and outgoing fluxes of FtsZ monomers must be balanced. Within our model this loss of FtsZ monomers from the ring occurs due to the hydrolysis of GTP followed by the rapid exclusion of the monomer; i.e. rate of GTP hydrolysis is coupled to the rate of the FtsZ monomer turnover. Thus, our model adheres to the “tight coupling” between GTP hydrolysis and FtsZ turnover observed in [32]. Assuming *k_hyd_* = 0.15 s^−1^ (reported by Romberg and Mitchison for *in vitro* conditions [13]), the model predicts a recovery half-time of about 6 seconds, somewhat shorter than the observed 30 seconds. This difference could be abolished by adjusting *k_hyd_* to 0.025 s^−1^, suggesting that there might be a difference between in *in vitro vs*. *in vivo* hydrolysis rates.

### Contraction of the Ring

**Figure 10:**
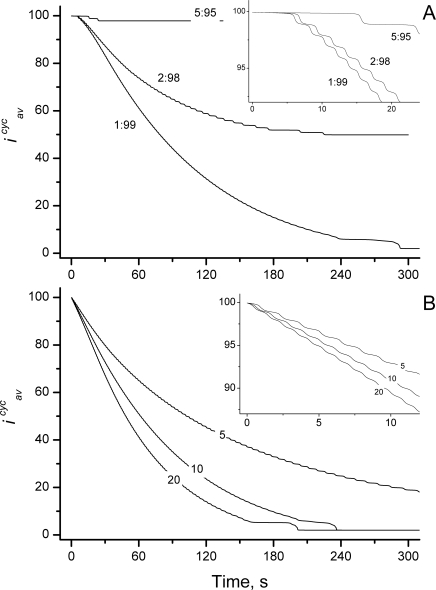
Contraction of Z Ring: Time Profiles of Average Ring Size *i^cyc^_av_*. (*A*) Constriction initiated by a shift in *GTP*/*GDP* ratio, with the value of *GTP*/*GDP* indicated. (*B*) Constriction initiated by the inhibitor controlled mechanism with a 5-fold (*k_nuc_*_1_ = *k_pol_*_1_ = *k_an_* = 0.8 µM^−1^ s^−1^), 10-fold (*k_nuc_*_1_ = *k_pol_*_1_ = *k_an_* = 0.4 µM^−1^ s^−1^) and 20-fold inhibition (*k_nuc_*_1_ = *k_pol_*_1_ = *k_an_* = 0.2 µM^−1^ s^−1^). Values at final points of the steady state simulations (calculated as in Fig. 9) were used as initial values; *i*_0_ = *i_av_^cyc^* was applied to calculate the rate of cyclization. All other parameters are as in Fig. 2 *C*–*D*. *Inset* shows kinetics on a shorter time-scale.

**Figure 11:**
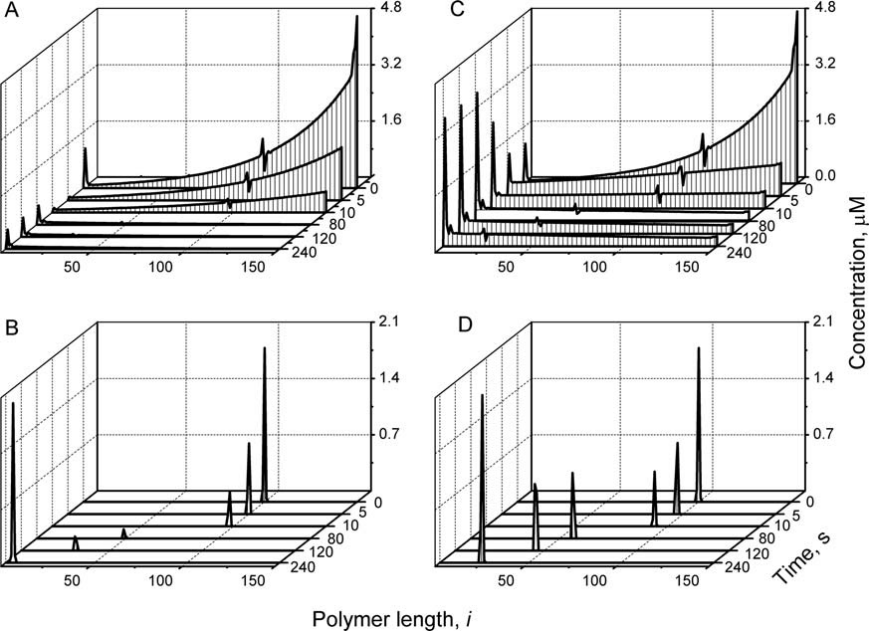
Contraction of Z ring. Polymer length distributions are plotted at different times. The concentration of polymers *Z_i_* are plotted *vs.* length *i* at different times. (*A*) Open polymers; (*B*) cyclized polymers for contraction initiated by the shift in *GTP*/*GDP* ratio: *GTP* = 1 µM, *GDP* = 99 µM. (*C*) Open polymers; (*D*) cyclized polymers for contraction initiated by the inhibitor controlled mechanism: *k_nuc_*_1_ = *k_pol_*_1_ = *k_an_* = 0.8 µM^−1^ s^−1^. Values at the final points of the steady state simulations (calculated as in Fig. 9) were used as initial values; the relationship *i*_0_ = *i_av_^cyc^* was applied to calculate the rate of cyclization. All other parameters are as in Fig. 2 *C*–*D*.

The dynamic steady state described above is indefinitely stable as long as the *GTP*/*GDP* ratio is invariant. Lowering the GTP/GDP ratio induced contraction of the ring within ∼5 min, with more dramatic ratio-changes causing more rapid contraction (Fig. 10 *A*). The corresponding changes in the distribution of open and cyclized polymers are shown in Fig. 11 *A* and *B*, respectively. An excess of GDP prevents polymer elongation due to an increase in inactive Z_D_ monomers. This shortens open polymers by hydrolysis/fragmentation in conjunction with the lack of active Z_T_ monomers available to restore long polymers (Fig. 11 *A*). This decrease in polymerization rate destabilizes rings and causes them to contract (Fig. 11 *B*).

This “metabolite controlled” contraction mechanism has two drawbacks. First, it appears that the *GTP*/*GDP* ratio is essentially constant in real cells [8]. Second, the total concentration of rings declines severely in the middle of the contraction process (Fig. 11 *B*). Although there are no quantitative experimental data on how the amount of FtsZ associated with the ring changes during contraction, we expect that the concentration of Z rings would not decline dramatically throughout this process.

Another approach to inducing contraction was to decrease polymerization rates, perhaps mimicking the effect of some hypothetical inhibitor. We simulated this by reducing the forward rate constants for nucleation, elongation and annealing (*k_nuc1_*, *k_el1_*, and *k_an_*). All three rate constants were inhibited to the same relative extents, as might be expected for a situation in which a hypothetical protein binds to the open ends of Z_T_ and to all Z_i_'s, thereby generating a form of FtsZ that is blocked from dimerizing, elongating and annealing. Reducing these rate constants 5-, 10-, or 20-fold induced ring contraction within a period of 2–5 minutes (Fig. 10 *B*), with the rate of contraction faster when the rate-constant-reduction was more severe. (The assumed absence of inhibition for cyclization could be viewed as an oversimplification. Thus, we have considered an expanded model with 2 annealing rate constants – one for cytoplasmic components (*k_an1_*) and one for membrane components (*k_an2_*). We assumed inhibition of the components exclusively in the cytoplasm by simultaneously reducing *k_nuc1_*, *k_el1_*, and *k_an1_*. In this case, the FtsZ ring contracted similarly to that reported with the original model.)

The time-dependent distributions of open and cyclized polymers for the case where rate constants were reduced 5-fold are shown in Fig. 11 *C* and *D*, respectively. As a result of this inhibition, a sizable proportion of FtsZ proteins become dimers. However, a significant portion remains as relatively long polymers, more so than by shifting the *GTP*/*GDP* ratio. This difference appears to be primarily responsible for the greater concentration of cyclized rings which remain during the contraction process (Fig. 11 *D*). This “inhibitor controlled” contraction mechanism retains Z rings at a higher concentration and thus is more likely to occur in real cells.

Although the rates of contraction were similar for the different mechanisms (Fig. 10, *A vs. B*), the shapes of the two plots were somewhat different. The plot arising from the inhibitor-controlled contraction mechanism decreased in a simple pseudo-exponential fashion (Fig. 10 *B*, *inset*) whereas that arising from the metabolite-controlled contraction mechanism was somewhat sigmoidal (Fig. 10 *A*, *inset*). Such differences might arise from the step within the mechanism which is controlled. In the metabolite-controlled mechanism, the nucleotide exchange reaction is perturbed, and it takes time for this perturbation to “work its way” through the system and influence ring size. In the inhibitor-controlled mechanism, the reactions directly involved in polymerization are perturbed, which more immediately influences ring size.

## Discussion

In this study, we have formulated a deterministic kinetic model for the assembly, dynamic maintenance, and contraction of the FtsZ ring. The model was designed at the biochemical level and with minimal complexity. By this we mean that the fewest reactions, components and assumptions necessary to achieve the desired behavior were used. Of the six reactions included, one serves to activate/deactivate FtsZ (GTP/GDP exchange), three reactions largely provide for the growth of polymers (nucleation, elongation, and annealing), one reaction is responsible for ring formation (cyclization), and one primarily functions in ring contraction but it also maintains the ring in a dynamic and responsive steady state (hydrolysis tied to the expulsion of FtsZ units from polymers). Once the Z ring is formed, its size becomes self-regulated. Simulations of the model were compared against pertinent experimental *in vitro* results and *in vivo* behavior. The overall kinetic behavior exhibited by the model simulations was semi-quantitatively similar to that observed for the assembly, stability, and contraction of the Z ring in *E. coli*. In our simulations, ring assembly occurred in seconds, whereas contraction required minutes, consistent with experiment. As we discuss below, a portion of the energy released during GTP hydrolysis is probably used to drive these processes.

### Design of the Biochemical Mechanism Assumed by the Model

The biochemical steps in the model were inspired largely by previous *in vitro* studies. Chen and coworkers [20] used a kinetic model of FtsZ assembly that included FtsZ activation (analogous to GTP/GDP exchange in our model), nucleation, and elongation. Their model was used to analyze fluorescence stopped-flow data for the *in vitro* assembly, in a physiological buffer, of an FtsZ mutant for which fluorescence was enhanced upon polymerization. Their model considered only polymers with lengths up to 7 subunits whereas our model includes three additional reactions and polymers as long as 150 monomeric units. Gonzalez *et al.* [35] considered a steady state model that included polymerization and cyclization reactions analogous to those included here. Neglecting hydrolysis and annealing steps allowed them to derive an analytical expression for polymer distribution at steady state. Based on a qualitative comparison of simulations to analytical ultracentrifugation sedimentation data, they concluded that the cyclization equilibrium constant is much stronger than that for polymerization. They also introduced the idea that FtsZ polymers have a flexible intrinsic curvature and that the probability of cyclization should depend on the length of the polymer *i*. Within our model we expand on these ideas by assuming that current membrane circumference dictates which open polymer length *i*_0_ is optimal for cyclization, and that the forward rate constant of the cyclization reaction is sensitive to polymer length.

In both previous kinetic and steady state models, GTP hydrolysis was neglected. As we have shown, this reaction is relevant for both the initial stage of assembly and at later stages as polymers elongate. Hydrolysis also influences the steady state system achieved, in terms of polymer distribution, the fraction of FtsZ that polymerizes, and the averaged length of filaments.

Annealing plays an important role in FtsZ assembly by generating long polymers. That this reaction occurs during FtsZ ring assembly had been proposed previously but it had not been included in any published biochemical mechanism. Annealing reflects the same fundamental tendency of FtsZ to self-association as is responsible for dimerization and elongation reactions. Thus, for consistency, annealing should be included (unless it is argued that annealing may be slower than these other processes and thus could be ignored). We found that annealing shifts polymer distributions *in vitro* toward longer polymers, which is important to achieve rings of appropriate size during *in vivo* assembly. Using AFM, Mingorance *et al.* [36] observed annealing and breakage of the FtsZ filaments *in vitro*, which demonstrates that these processes should be included in models.

With the chosen rate constants, our model predicts the same effect of GTP hydrolysis on the critical concentrations for FtsZ assembly close to those reported, namely an increase in critical concentration with faster hydrolysis [19],[21]. It also predicts a shift in the average size of FtsZ polymers with changes in the rate of hydrolysis similar to that measured by the Erickson group [12]. Mimicking this shift qualitatively required both hydrolysis and annealing steps. By shutting off the hydrolysis reaction *in silico*, our model qualitatively reproduces the kinetics of stopped-flow experiments that were carried out in the absence of hydrolysis (in buffer lacking Mg). The model predicts that the system reaches steady state slower in the absence of the hydrolysis reaction, similar to the results and conclusion of the original works.

### Additional Features and Assumptions for Modeling the Z Ring *In Vivo*

After successfully testing the model using available *in vitro* data, we applied exactly the same biochemical mechanism, including the same rate constants, to the analogous *in vivo* situation. A number of additional assumptions were required for an *in vivo* setting, the most important of which was that the Z ring could assemble only within a narrow mid-zone region of a cell membrane. Our current model does not include a mechanism for such localization, but this is thought to involve the MinCDE system and/or nucleoid occlusion [2],[3],[6]. Nor does our model consider the mechanism that controls the *timing* of cytokinesis events, including the assembly, steady state maintenance, and contraction of the Z ring. We suppose that features of FtsZ alone determine the dynamics of the Z ring, while progression from one phase to another is independently controlled. As such we have not modeled the commitment from one phase to the next. Rather, we modeled each phase of ring dynamics separately. Indeed, our results show that the FtsZ system alone might be responsible for the observed kinetics of assembly and contraction. Our model implies that such control might be realized by an inhibitor (or several inhibitors) of polymerization. Then, a drop in the concentration of such an inhibitor might lead to Z ring assembly. A subsequent increase in the inhibitor concentration (or in the concentrations of other similarly-acting species) might induce ring contraction. Lastly, our model does not include the final step in cytokinesis – namely cell scission. There are probably additional processes required to accomplish this.

A third major assumption of our model is that FtsZ polymers of lengths greater than dimers are attached to the membrane, which in reality, might be realized through interactions with other proteins such as FtsA and/or ZipA [1],[6],[8]. Since we did not consider mechanisms of attachment explicitly, we simply assumed that all trimers and higher polymers are surface-bound. To assess whether this assumption of a “critical” length for membrane attachment is important in the behavior of the model, we performed simulations of a modified model in which only pentamers and higher polymers were attached to the membrane and shorter polymers were localized to the cytosol; no differences in qualitative behavior were observed with only minor changes in quantitative behavior (*i.e.* the cytosolic fraction was shifted from 20% to 22%).

A fourth set of assumptions in our model involved the cyclization reaction. We assumed that the rate constant for cyclization varies with the length of the reacting open polymer. Prior to Z ring formation, the optimal length for cyclization (*i*_0_) is a fixed parameter external to the dynamical system and dictated by the circumference of the membrane. After the Z ring (or rings) form(s), Eq. 5c is applied, such that the optimal length for the synthesis of new Z rings *i*_0_ is dictated by the average size of existing Z rings, which in turn is dictated by the dynamics of the system. Thus, after the ring assembly, *i*_0_ becomes an internal parameter for the FtsZ system – i.e. controlled by the current state of the system.

The combination of Eq. 5b with 5c prevents the optimal cyclization length *i*_0_ from changing dramatically when a single Z ring opens. There are several possible mechanisms which could provide this. Firstly, cyclization might be much faster than the time of the membrane remodeling. Secondly, several Z rings could form at any given moment, and one such ring could open (and shorten) while the remaining rings maintain the membrane at the current circumference. A third mechanism involves cell wall synthesis. If the FtsZ ring opens, the rigid cell wall could keep the membrane at a relatively stable circumference. Once the FtsZ ring opens and incrementally squeezes, the cell wall would remodel to the new circumference. If this last mechanism is realized, the stable steady state required in the FtsZ model might not be necessary. This would be the case since Eq. 5c is only valid when the cell wall synthesis machinery is assembled, which may permit ring contraction. However, this cell-wall-remodeling mechanism seems less likely since there are wall-less cells - *viz*. Mycoplasma - which contain an FtsZ homolog and almost certainly divide by a mechanism similar to that used by other FtsZ-containing cells.

We used a Gaussian function to describe the *i*-dependence of *k_cyc_*(*i*) implying a symmetric minimum in the chemical potential around *i*_0_. We considered an asymmetric potential as a possible expansion of the model, but this did not change the qualitative behavior of the system significantly, although it helped to stabilize self-regulated rings.

### Model Simplicity

We tried to keep our model of the Z ring as simple as possible. Since such a minimal model exhibits the desired behavior, extra features that are not included in the model appeare to be unnecessary in generating this behavior. One simplifying assumption was that Z_D_ monomers do not polymerize. Under some experimental conditions, GDP-bound FtsZ polymers have been observed [18],[19]. However, the affinity between units was much less than for GTP-bound units, which resulted in very short polymers. This suggests that GDP-bound FtsZ polymers may not have any physiological significance, and so we have ignored this type of polymer in our model. Consistent with this, we also assumed that any FtsZ polymer containing a GDP-bound unit will fragment as a Z_D_ unit is expelled.

Another simplification of our model involved neglecting GTP/GDP exchange within assembled FtsZ polymers. In contrast to the tubulin dimer structure, nucleotides bound within the FtsZ dimer are not completely occluded by the protein surface [14]. On this basis, it has been proposed that the opening is large enough for nucleotides to pass into and out-of the active site, allowing GTP/GDP exchange to occur in FtsZ polymers. We examined the potential importance of this polymer-level GTP/GDP exchange reaction by expanding our current model (Text S1). This expansion did not lead to any major additional critical behavior (data not shown), though it does allow us to explain the observed coupling between rates of GTP hydrolysis and FtsZ subunit turnover (see below).

Another simplifying assumption of our model was that χ was considered to be a constant. In the future, we plan to relax this assumption by incorporating our FtsZ model within a growing and dividing whole-cell model [38]. Nor does our model consider bundling or overlapping of FtsZ filaments. There is a modicum of experimental evidence for bundling, but no functional properties have been established. There has been speculation that such bundles cause cooperative behavior and that they might be involved in the mechanism of contraction. Our results show that bundling or overlapping is not required for Z ring constriction *per se*.

### Limitations of the Model

In this study, we have described the chemical kinetics of the FtsZ system by applying deterministic equations based on the law of mass action which is valid when reacting species are abundant. To make *quantitative* predictions of behavior an *in vivo* kinetic model should take into account low copy-numbers of species present in real cells and the confined space of real cells. Using deterministic kinetics allowed us to simplify the problem relative to a stochastic treatment, but it should be viewed as a “stepping stone” toward a more comprehensive quantitative model of Z ring dynamics based on a stochastic treatment. Such treatments use deterministic rate constants as inputs in estimating probabilities of reactions. With a viable set of values in hand, it should be possible to “stochasti-size” our model and determine whether the modeling approach (deterministic *vs.* stochastic) influences the stability of the rings, alters the polymer distribution, or modifies dynamical behavior. Another consideration would involve the introduction of fractionary-order kinetics due to interfacial reactions and/or macromolecular crowding [43].

Although many of the kinetic parameters used in this model have been estimated from experimental results, others were adjusted so as to achieve desired behavior. Through this process, we have noticed that more than one set of parameters, when used in conjunction with our model, is able to achieve the same desired qualitative behavior. Thus, our model and parameter set are not unique. Further restrictions of these parameters will require fitting to additional experimental results and perhaps model expansion.

We applied quasi steady state conditions to all intermediates of the form {Z_j_Z_d_Z_i−j−1_} (Fig. 1 R6). This allowed us to eliminate such species from ODEs and to avoid considering all polymers with higher number of GDP-bound FtsZ units in the chain (such as Z_j_Z_d_Z_d_Z_i−j−2_). This is a simplification of reality, but it made our analysis possible. Also, the collective experimental evidence suggests that this simplification does not deviate much from reality. As mentioned above, GDP-bound FtsZ polymers disassemble quickly [6], and Romberg and Mitchison determined that about 80% of FtsZ in polymers are in the GTP-bound form [13]. There is also some evidence that GTP hydrolysis is the rate-limiting step in the hydrolysis/fragmentation process [13],[14].

The last limitation of the model considered here is that *i_max_* = 150. This value was dictated by the limitations of the software used for simulations. Even with this maximal value, the model includes 300 ODEs plus a number of algebraic equations. The major drawback of this assumption is that the optimal *i*_0_ = 100 will result in a ring size about 7 times shorter than in real *E. coli* cells. For *in vitro* simulations *i_max_* is sufficient for quantitative simulations, since most of the observed polymers are generally shorter that this maximal value. As a consequence, our results for *in vitro* modeling can be compared quantitatively to experimental results, whereas those for *in vivo* modeling can only be compared qualitatively.

### Model Behavior

**Figure 12:**
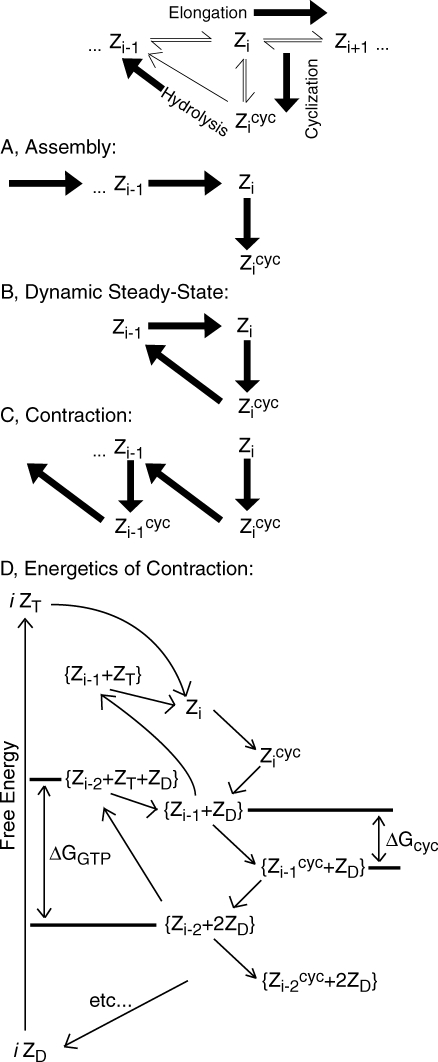
Flux and Energy Dynamics Associated with Model Behavior. (*A*) Assembly: long open FtsZ polymers are produced by elongation, which then cyclize into closed ring structure – Z ring. (*B*) Dynamic steady state: hydrolysis followed by fragmentation leads to opening of the ring which is then restored by addition of one monomer followed by cyclization. Also ring appears to be stable, with constant breaking/closing of the ring in the system. (*C*) Contraction: hydrolysis again results in an open polymer one subunit shorter, but since elongation is inhibited now, this is followed by cyclization at a suboptimal polymer length. This results in a ring one subunit shorter than before this process. (*D*) Energetics of contraction: the energy of GTP hydrolysis is released in several steps, including self-assembly into open polymers, cyclization resulting in closed structures, and expulsion of GDP-FtsZ from polymer chains. Cyclization is coupled to membrane constriction.

The model presented here exhibits the qualitative behavior observed regarding the assembly, dynamic stability, and contraction of the Z ring. This behavior can be simplistically understood by considering the dynamics of the ring as arising from a balance between hydrolysis, cyclization and elongation reactions, as illustrated in Fig. 12. During assembly, the dominant flux through the system is L-shaped in this figure, with the position of the short leg of the L corresponding to the selected optimal ring circumference *i*_0_ (Fig. 12 *A*). After assembly, when the position of most favorable cyclization is free to vary, the size of the ring is stable when these three reactions are balanced and the dominant flux forms a triangular pattern (Fig. 12 *B*). By increasing the cyclization rate (or by decreasing the polymerization rate), the system shifts such that the dominant flux forms a saw-tooth pattern which results in ring contraction (Fig. 10 *C*). A new steady state can be achieved at shorter ring sizes because the effective rate of hydrolysis for a given polymer is proportional to *i*, which counterbalances the increased rate of cyclization.

Fig. 12 underlines the highly dynamic nature of the steady state condition, where ring cleavage and cyclization occur continuously, as was assumed in explaining the FRAP experiments [32],[33]. These interrelationships can explain how the rate of GTP-hydrolysis is connected with FtsZ subunit turnover. As mentioned in *Results*, an inherent feature of the presented model is the strong coupling between these two processes (when a decrease in *k_hyd_* leads to a decline in FtsZ turnover to the same extent), in agreement with observations [32]. Subsequently, Anderson *et al.* [33] measured a 9 second turnover time in *E. coli* for wild-type FtsZ and a 30 second turnover time for the FtsZ84 mutant. Although the value of 9 seconds for WT is closer to the 6 seconds predicted by the model (assuming *k_hyd_* = 0.15 s^−1^) than to the previously reported 30 seconds, these results suggest that the level of coupling between GTP hydrolysis and subunit turnover is variable and is not as tight as previously assumed (a 10-fold decrease in *k_hyd_* leads to only a 3-fold decrease in FtsZ turnover). Such “loose” coupling can be explained by an expanded model which includes nucleotide exchange reactions in polymers (see Text S1). With these reactions included, there is no requirement that every GTP hydrolysis event must lead to polymer fragmentation since GTP can substitute for GDP within unstable intermediate Z_j_Z_D_Z_i−j−1_ polymers. This substitution stabilizes the polymer and prevents fragmentation, resulting in loose coupling. Moreover, using the expression for fragmentation rate given in Eq. A.7 (Text S1), the ratio of rates for hydrolysis and exchange in polymers can be estimated. Assuming *k_FRAP_* = 0.07 s^−1^ and 0.023 s^−1^ for WT and FtsZ84, respectively, we estimate that *k_hyd_*/(*k_ex2_ GDP*)≈3. For the earlier FRAP experiments of Sticker *et al.* [32], the same analysis suggests *k_hyd_*/(*k_ex2_ GDP*)≈90. In either case, it appears that rate of GTP hydrolysis in polymers dominates relative to that of nucleotide exchange.

### Alternating Rates of Contraction

Perceptive readers will have noticed that the average ring circumference declines in a step-wise manner, revealing a jagged saw-tooth pattern in both Fig. 9 and Fig. 10. This pattern arises because the rate of contraction exhibits a periodic auto-acceleration, ultimately due to the narrow distribution of *k_cyc_*(*i*) and the discrete nature of the polymer distribution for the Z_i_'s. At any given time, only ∼3 open polymers participate significantly in cyclization, including the polymer closest to *i^cyc^_av_* and the polymers one subunit longer or shorter (see inset of Fig. 2 *D*). If *i^cyc^_av_* is close to a natural number (e.g. *i^cyc^_av_*≈100), then cyclization of the corresponding Z_i_ (i.e. Z_100_→Z^cyc^_100_) dominates, and the system tends to maintain the same *i^cyc^_av_*. However, if *i^cyc^_av_* is staggered between natural numbers (e.g. *i^cyc^_av_*≈99.5), there are two equally strong cyclization processes (Z_99_→Z^cyc^_99_ and Z_100_→Z^cyc^_100_). Since hydrolysis of the Z ring always leads to an open polymer one subunit shorter than the ring (Z^cyc^_100_→Z_99_), this will shift the flux from bigger rings to smaller ones (Z^cyc^_100_→Z^cyc^_99_), which will cause further changes in *i^cyc^_av_*. So, the decline in *i^cyc^_av_* leads the system from a metastable state (*i^cyc^_av_*≈100) to a non-stable state (*i^cyc^_av_*≈99.5), until *i^cyc^_av_* approaches the next natural number (*i^cyc^_av_*≈99), in which case the system returns to a metastable state. The process repeats to afford the observed pattern in Fig. 9 and Fig. 10.

### Advantages of the Model

Although our kinetic model is based on previous models describing FtsZ assembly, it extends well beyond these earlier models. Specifically, ours is the first to include not only FtsZ ring assembly as a behavioral target, but also dynamic stability and contraction. It may also be the most tested model of its kind, in that we have tried to simulate all pertinent published results using it. Our simulations mimic these results with various degrees of fidelity, ranging from highly qualitative to semi-quantitative.

That the model was constructed to be of minimal complexity is also significant, because it is now clear that no reactions besides the six that we have included are needed to achieve the behavior described here. Including other reactions will be required to mimic more complex behavior (e.g. how the ring is positioned and attached to the membrane) or to regulate the transition from one state of the ring to the next (i.e. assembly → stability → contraction), but doing so is beyond the scope of the current study.

The model is the first to consider both *in vitro* and *in vivo* settings for these processes, and an ability to switch easily between these settings. We used the same set of rate constants for both settings. Quantitative biochemical information is generally available only for *in vitro* conditions. This raises the issue of whether this information can be transferred to *in vivo* conditions. The successful *in vivo* modeling of FtsZ behavior as demonstrated here, using the same mechanism and kinetic parameters, suggests that this is indeed the case.

With our approach, we need only change χ and σ to switch between *in vitro* and *in vivo* settings. χ is the surface-to-volume ratio which must be included in rate expression terms for particular species that are involved in interfacial reactions. In a previous study we found that these modifications of rate law expressions are required to accurately model interfacial reactions [38]. In real cells, the processes of FtsZ ring assembly, stability and contraction *must* include interfacial reactions, since the monomeric units of the ring are synthesized by ribosomes in the cytosol whereas the ring itself and undoubtedly some (or all) open polymers are surface-bound. The unknown issue here is the step of the process for which interfacial reactions should begin. In our model, we assume that monomer and dimer FtsZ units are volume-bound whereas trimers and longer FtsZ polymers are surface bound.

The importance of interfacial considerations is highlighted by the very different behavior observed when *in vitro* vs. *in vivo* conditions are assumed. When *in vivo* conditions are assumed, the distribution of open polymers favors longer polymers and the concentration of cyclized polymers is dramatically higher. This difference arises due to the small value of χ assumed, which leads to an “effective” increase in the concentrations of polymers on the surface mid-zone. It is generally tempting to attribute any and all differences between *in vitro* vs. *in vivo* behavior to the presence of additional (often unknown) proteins or metabolites under *in vivo* settings and their absence under *in vitro* conditions. Our study suggests that some observed differences in behavior may simply arise from differences in the *geometry* of the system. With regard to our particular model, the possibility that FtsZ polymers which are assembled *in vitro* may actually involved interfacial reactions (e.g. polymers may form exclusively on the surface of the reaction vessel) should also be considered. If this were the case, reported rate constants may need to be recalculated.

### Driving Force of Z Ring Contraction

Many issues remain regarding cytokinesis in prokaryotes, including identifying the force which drives membrane constriction, describing mechanistically how the Z ring functions during this process, and the overall role of the Z ring in membrane constriction. Several mechanisms of Z ring contraction have been proposed [2],[3],[6]. Analogous to those of the eukaryotic contractile ring, relatively short FtsZ filaments may slide against each other without disassembling, driven by an unidentified motor protein. Another possibility is that the bending of FtsZ filaments (resulting from the hydrolysis of GTP) drives constriction. Alternatively, FtsZ subunits may be expelled from the ring without loss of ring integrity, thereby causing constriction. Finally, a pinching force provided by inward growth of the septum wall may be involved in constriction. In this case, the Z ring would passively contract ahead of the wall invagination and would serve only as a scaffold protein for the divisiome machinery. Although our model is compatible with the latter two cases, we suggest below another mechanism of contraction.

Until recently, the sliding model has been most popular despite the fact that no motor protein associated with the Z ring has been identified [44]. Osawa and Erickson recently demonstrated that FtsZ from divergent species can successfully operate in *E. coli*, despite low sequence identity [45]. Although their results did not eliminate the sliding mechanism completely, they made it unlikely. Osawa and Erickson suggest that a hypothetical motor protein is unnecessary and that “FtsZ may generate the constriction force through *self-interactions*, not requiring interaction with downstream cell division proteins”. Our model supports their suggestion, as it does not include other proteins (motoring or otherwise) yet the Z ring can contract. Our model provides an explicit biochemical mechanism which could serve as a possible set of “self-interactions”.

What drives contraction within our model is the high affinity of GTP-FtsZ to self-assembly which results in a non-zero probability of cyclization at suboptimal length. GTP hydrolysis opens and shortens the ring by one subunit. There are two main scenarios regarding what will happen with this open ring. Elongation by one subunit followed by rapid cyclization will restore the initial ring (Fig. 12 *B*). Alternatively, immediate cyclization after ring opening will result in a ring one subunit smaller then the initial one (Fig. 12 *C*). The probability of such suboptimal cyclization is non-zero, although it is lower than that for cyclization of an optimally-sized polymer. This is so because cyclization requires membrane remodeling, and thus is energetically suppressed (Fig. 12 *C*). These two alternative pathways are competing, and at steady state addition of GTP-FtsZ monomers is more likely which leads to the appearance of a stable ring. Once the probability of monomer addition is suppressed (i.e. by lowering GTP/GDP ratio or by inhibition of *k_el_*), this balance between hydrolysis/elongation/cyclization is upset, and suboptimal cyclization dominates which results in ring contraction.

Cyclization occurs when the free ends of the polymer contact each other due to thermal motion. Eq. 5c with the distribution for the forward cyclization rate (meaning also the distribution for *K_cyc_*) implies that the conformation of polymers in which open ends are in contact are less favorable for polymers of non-optimal length. But once these ends are close to each other, the probability to form a closed structure is high. The probability of finding open ends in proximity depends on two factors: the distance between the ends and work necessary to bend the membrane and pull the two ends close together. In this study, we considered two distributions for cyclization rate, including σ = 10 (*in vitro*) and σ = 0.5 (*in vivo*). Although forces are not considered explicitly in our model, using the narrow distribution for the *in vivo* case implies that there is work used for membrane bending. However, this distribution is also influenced by a geometrical factor describing changes in available conformations of the ring, such that the work contribution cannot be quantified.

Such a process of Z ring contraction utilizes the energy of GTP hydrolysis indirectly to constrict the membrane. The energy of each GTP hydrolysis event is released in several steps. Initially, energy is stored in the form of energetically enriched GTP-FtsZ (Z_T_ compared to Z_D_, with Δ*G_GTP_* representing the difference in energy per molecule of FtsZ, Fig. 12 *D*). A portion of Δ*G_GTP_* is released in a spontaneous elongation reaction, when Z_T_ units attach to a growing open polymer. Another portion is released when the polymer cyclizes. A Z ring of length *i* can be viewed as being “high-energy” relative to *i* Z_D_ units but lower energy than *i* Z_T_ units (Fig. 12 *D*). What remains of Δ*G_GTP_* is released when GTP is hydrolyzed to GDP and a Z_D_ unit is expelled (Fig. 12 *D*). Only the portion of Δ*G_GTP_* which is released in cyclization at suboptimal length is coupled to unfavorable membrane constriction (Fig. 12 B), and thus is the chemical force of contraction within this scheme. Another critical role of GTP hydrolysis is to make the ring unstable which together with the changes in the conditions (with decreased probability of elongation) provides the direction for changes in ring size. Cyclization “locks” the new conformation of the rings which then becomes the current state for the system. The process repeats until ring contraction ceases.

Given the equilibrium constant for the cyclization reaction used in our simulations (*K_cyc_* = 150) the apparent force provided by such a mechanism can be estimated. The free energy change is Δ*G_cyc_* = −*k_b_T*ln*K_cyc_*≈2×10^−20^ J. (*k_b_* and *T* are Boltzmann gas constant and absolute temperature with values 1.38×10^−23^ J/K and 300 K, respectively). One reaction event would shorten a ring by one subunit, thus the maximum force generated would be *F* = Δ*G_cyc_*/*d*≈5 pN (*d* = 4 nm is the length of a monomer). This is a lower limit of the force available for contraction, since we used a modest value of *K_cyc_* (compared to 10^4^ assumed in [35]). Recently, Lan *et al.* [37] considered the mechanics of cytokinesis for bacteria with a cell wall. By considering the material parameters of the *E. coli* cell wall, they concluded that a force as small as 8 pN would be sufficient to enforce contraction. Within their model a small force provided by the ring leads to incremental invagination of the plasma membrane which is then followed by remodeling of a rigid cell wall. They assumed a constant force generated by the Z ring, but did not detail the molecular origin of that force. The model presented here provides a molecular mechanism of how the known chemical features of the FtsZ protein, including GTPase activity, could result in such a force. Besides indicating forces of the same magnitude, the time of contraction exhibited by their model (∼300 s) is similar to those reported here. Thus, these two models are *absolutely compatible* in describing prokaryotic cytokinesis, with the model of Lan *et al.* treating the mechanics of contraction and our model treating the chemical mechanism.

### Conclusion

The study presented here demonstrates that currently known biochemical reactivity and physical properties of the FtsZ protein (as revealed by *in vitro* studies) are sufficient to afford observed *in vivo* behavior. Such behavior includes the assembly of the Z ring, maintenance of the ring in a dynamic steady state, and contraction of the ring. Within this minimal system, Z ring contraction does not require motor proteins and is driven by the strong tendency of GTP-bound FtsZ proteins to self-assemble. This model provides a foundation upon which a more comprehensive understanding of bacterial cytokinesis can be investigated.

## Supporting Information

Figure S1 Polymer distributions *in vitro* as a function of total FtsZ concentrations. Total concentration of open and cyclized polymers Z*^cyc^_i_ +* Z*_i_* are plotted *vs.* length *i* at the end of the assembly. (*A*) *k_hyd_*  =  0.15 s^-1^; (*B*) *k_hyd_*  =  0.0 s^-1^. Other parameters as in Fig. 2 *A* and *B* except that Z*_tot_*  =  10 µM. Polymer distributions presented in *B* and their dependence on total FtsZ concentration are qualitatively similar to sedimentation distribution results reported by Gonzalez *et al.* ([35], Fig. 1C).
(186 KB TIF)

Figure S2 Changes in polymer distributions *in vivo* after assembly. Concentrations of open (*A*) and cyclized (*B*) polymers are plotted *vs*. length *i* before Eq. 5c applied (*blue*) and after system reach new steady state (*red*). Parameters as in Fig. 2 *C–D*, except *k_cyc_* =  100 s^-1^. Simulations were performed as in Fig. 9. Note, that initial and final distributions for open polymers are almost indistinguishable except narrow zone around optimal cyclization.
(158 KB TIF)

Text S1 Model with nucleotide exchange in FtsZ polymers.
(36 KB DOC)
