# Supplementary material for: Kinetic Modeling of the Assembly, Dynamic Steady State, and Contraction of the FtsZ Ring in Prokaryotic Cytokinesis
Source: PLoS Comput Biol. 2008 Jul 4;4(7):e1000102. doi: 10.1371/journal.pcbi.1000102 (PMC2432035; doi:10.1371/journal.pcbi.1000102)
Supplement: Text S1 — Model with nucleotide exchange in FtsZ polymers. (36 KB DOC) [file pcbi.1000102.s003.doc]

**SUPPLEMENTAL MATERIAL**

to "Kinetic Modeling of the Assembly, Dynamic Steady State, and Contraction of the FtsZ Ring in Prokaryotic Cytokinesis" by Surovtsev I.V., Morgan J.J., and P.A. Lindahl

**Model with nucleotide exchange in FtsZ polymers.**

The main model described in the text was expanded to include the following GTP/GDP nucleotide exchange reaction within FtsZ-polymers:

(A.1)

The notation used here is the same as for nucleotide exchange in monomers (Fig. 1, *Reaction 1*), where the replacement of GDP in polymers with GTP is considered as being the forward direction. According to the assumptions of the model, the Z_j_Z_D_Z_i-j-1_ intermediate is a result of GTP hydrolysis (Fig. 1, *Reaction 6*) and is unstable, such that Z_D_ exclusion follows immediately. Forward exchange of GDP to GTP returns Z_j_Z_D_Z_i-j-1_ to the stable Z_i_ state, and thus prevents FtsZ monomers from being expelled, and thus stabilizing long FtsZ polymers. In turn, the reverse exchange of GTP to GDP within polymers leads to Z_j-1_Z_D_Z_i-j-1_ formation and thus to destabilizing long FtsZ polymers.

Due to the common intermediate Z_j_Z_D_Z_i-j-1_, GTP/GDP exchange in polymers (A.1) and the hydrolysis reaction (Fig. 1, *Reaction 6*) are connected, and both are followed by fast Z_D_ expulsion:

(A.2)

Similar to our previous considerations, we assume that *Z_j_Z_D_Z_i-j-1_* is a short-lived intermediate for which we apply the quasi-steady-state approximation. This allows us to substitute *Z_j_Z_D_Z_i-j-1_* with constant algebraic expressions in the kinetic equations and not to consider higher intermediates such as Z_i-2_Z_D_Z_D_, Z_i-3_Z_D_Z_D_Z_D_ *etc*. This leads to two effective reactions, namely GTP-ase and fragmentation reactions, respectively:

, (A.3)

; (A.4)

Thus, Z_i_ becomes a catalyst for the hydrolysis of GTP to GTP. The effective rates for open and cyclized polymers, respectively, are as follows:

$R_{fr}^{i}=k_{dis}\frac{k_{hyd}(i-1)+k_{ex2}^{pol}\cdot i\cdot GDP}{k_{dis}+k_{ex1}^{pol}\cdot GTP} Z_{i}$, (A.5)

$R_{GTPase}^{i}=k_{ex1}^{pol}\frac{k_{hyd}(i-1)+k_{ex2}^{pol}\cdot i\cdot GDP}{k_{dis}+k_{ex1}^{pol}\cdot GTP} GTP Z_{i}$, (A.6)

$R_{fr\_cyc}^{i}=(k_{hyd}+k_{ex2}^{pol}\cdot GDP)\frac{k_{dis}}{k_{dis}+k_{ex1}^{pol}\cdot GTP}i Z_{i}^{cyc}$ (A.7)

$R_{GTPase\_cyc}^{i}=(k_{hyd}+k_{ex2}^{pol}\cdot GDP)\frac{k_{ex1}^{pol}}{k_{dis}+k_{ex1}^{pol}\cdot GTP} i GTP Z_{i}^{cyc}$, (A.8)

Reaction (A.3) unites two processes, including hydrolysis and GTP/GDP exchange reactions followed by Z_D_ expulsion. This leads to the same products, including Z_D_ and 2 short polymers Z_j_ and Z_i-j-1_. Eq. A.4 describes the apparent GTPase activity of FtsZ polymers, when GTP hydrolysis is followed by GDP/GTP exchange and thus does not change polymer length. Hence, this GTPase reaction does not change the concentration of certain polymers - it changes only the concentration of GTP and GDP, and thus does not lead to new terms in the kinetic equations.
